# The efficiency and effectiveness of utilizing diagrams in interviews: an assessment of participatory diagramming and graphic elicitation

**DOI:** 10.1186/1471-2288-8-53

**Published:** 2008-08-08

**Authors:** Muriah J Umoquit, Mark J Dobrow, Louise Lemieux-Charles, Paul G Ritvo, David R Urbach, Walter P Wodchis

**Affiliations:** 1 Cancer Services & Policy Research Unit, Cancer Care Ontario, Toronto, ON, Canada; 2 Department of Health Policy, Management and Evaluation, University of Toronto, Toronto, ON, Canada; 3 School of Kinesiology and Health Sciences, York University, Toronto, ON, Canada; 4 Department of Psychology, York University, Toronto, ON, Canada; 5 Division of Preventive Oncology, Cancer Care Ontario, Toronto, ON, Canada; 6 Department of Public Health Sciences, University of Toronto, Toronto, ON, Canada; 7 Division of Epidemiology, Biostatistics and Behavioural Sciences, Ontario Cancer Institute, University Health Network, Toronto, ON, Canada; 8 Department of Surgery, University of Toronto, Toronto, ON, Canada; 9 Toronto Rehabilitation Institute, Toronto, ON, Canada; 10 Institute for Clinical Evaluative Sciences, Toronto, ON, Canada

## Abstract

**Background:**

This paper focuses on measuring the efficiency and effectiveness of two diagramming methods employed in key informant interviews with clinicians and health care administrators. The two methods are 'participatory diagramming', where the respondent creates a diagram that assists in their communication of answers, and 'graphic elicitation', where a researcher-prepared diagram is used to stimulate data collection.

**Methods:**

These two diagramming methods were applied in key informant interviews and their value in efficiently and effectively gathering data was assessed based on quantitative measures and qualitative observations.

**Results:**

Assessment of the two diagramming methods suggests that participatory diagramming is an efficient method for collecting data in graphic form, but may not generate the depth of verbal response that many qualitative researchers seek. In contrast, graphic elicitation was more intuitive, better understood and preferred by most respondents, and often provided more contemplative verbal responses, however this was achieved at the expense of more interview time.

**Conclusion:**

Diagramming methods are important for eliciting interview data that are often difficult to obtain through traditional verbal exchanges. Subject to the methodological limitations of the study, our findings suggest that while participatory diagramming and graphic elicitation have specific strengths and weaknesses, their combined use can provide complementary information that would not likely occur with the application of only one diagramming method. The methodological insights gained by examining the efficiency and effectiveness of these diagramming methods in our study should be helpful to other researchers considering their incorporation into qualitative research designs.

## Background

Health care systems around the world are increasingly interested in the use of financial incentives to improve quality of care and system performance[1,2]. However, there is considerable uncertainty about how these 'pay-for-performance' or 'value-based purchasing' models impact on clinical behaviour. Therefore, we initiated a qualitative research study to examine how financial incentives affect clinical behaviour within the context of the cancer system in Ontario, Canada.

From a study design perspective, a critical challenge was how to efficiently and effectively collect data on the multifaceted and diverse nature of clinical accountability relationships through key informant interviews with clinicians and senior administrative leaders. Communication of questions and answers may be more clearly expressed when a visual aid is available for both interviewer and interviewee to reference[3]. This is especially applicable when dealing with highly complex or sensitive topics which are difficult to communicate about fully through strictly verbal exchanges[4]. As key informant interviews often deal with such topics and require participants to articulate personal opinions and explain complex processes or relationships, the use of diagrams within an interview setting is one way to facilitate the data collection process, providing benefits to both the interviewer and the interviewee. This paper focuses on two diagramming methods designed to foster the extraction of high quality research data via interview. In the first method, 'participatory diagramming', respondents create a diagram to assist in their responses to interview questions. In the second method, 'graphic elicitation', a researcher-prepared diagram is used as a stimulus for gathering information from interviewees. These two methods come from different academic backgrounds and have unique benefits and challenges, however their common purpose is the efficient extraction of rich and accurate data.

While participatory diagramming and graphic elicitation have been shown to offer benefits to researchers, there has been no systematic evaluation to guide in their application. As part of our larger qualitative research study investigating how financial incentives affect clinical behaviour in Ontario's cancer system, the two diagramming methods were applied in over 60 key informant interviews. Using both quantitative criteria and qualitative observations, we assessed both the efficiency and effectiveness of these respective methods for collecting data and facilitating analyses. It is our hope that this paper will expose and describe the strengths and weaknesses of these two diagramming methodologies to a wider audience and be a useful guide for future qualitative research.

### Participatory diagramming

Participatory diagramming is a term that refers to a set of research techniques and includes a variety of diagramming methods including the use of timelines, flowcharts and/or tables [5-7]. Often this method is used in a focus group setting, as part of participatory action research methodologies, where subjects being studied are encouraged to simultaneously contribute to the derivation and analyses of data[5,8]. For the purpose of this study we used participatory diagramming strictly as a data collection method rather than as a broader research methodology as it was originally intended[5,7]. While there are multiple ways to incorporate participatory diagramming methods in a research design, the common element requires the respondent to create a visual interpretation of the topic of interest, depicting the relevant components and inter-relationships[9].

The work of Kesby[5,10,11] provides key examples of the utilization of participatory diagramming in data collection. Working with adults in rural Africa, Kesby facilitated the creation of flow and matrix diagrams to assist in the discussion of sensitive topics and to overcome cultural and language barriers. In addition, participatory diagramming has been effectively used to break down communication and social barriers between street-kids and researchers[12]. Even where such barriers do not exist, as in White's[13] study of an American college's administrative processes, the creation of a diagram by interviewees can be beneficial to data collection. Creating a visual diagram can help key informants articulate concepts and ideas, while leaving the researcher with a physical product that reflects the participant's own priorities and interests[7]. The challenges that come with using this method revolve mainly around participants' varying comfort levels and abilities to visually depict ideas in a coherent manner. Not everyone is confident in their ability to create a diagram, especially in an interview setting where time is limited and they may have the perception that there is insufficient time to prepare[7].

### Graphic elicitation

Graphic elicitation in interviews involves presenting interviewees with researcher-prepared visual stimuli. The prepared visual aid encourages dialogue and/or a reaction and elicits the interviewee's perspective relative to the visual stimulus[14]. The visual stimulus is typically informed by previous research and therefore has the potential to be used as a form of research validation. While this method facilitates the collection of data, questions remain about the level of accuracy, the appropriate ages to which this method should be targeted and the potential for introducing bias into the data collection process[15,16].

Crilly et al. provide a well-documented case of graphic elicitation being effectively used in interviews[3,4]. They employed multiple diagrams in interviews with industrial designers to understand the factors and processes that influence product appearance. They found that prepared diagrams acted as a reference point for discussion, allowing the elicitation of information that may not have been obtained through questioning alone. They concluded that graphic elicitation was both a practical and effective data collection technique given their sample and research goals[4]. However, it has been acknowledged that prepared diagrams have the potential to influence or restrictively bias interviewees' thinking, rather then helping to stimulate, expose or reflect it[4,16]. Furthermore, diagrams are most useful to those who have the ability and skill to quickly interpret them[17]. Crilly et al. have focused their work on the design industry and have stressed that studies across other populations are needed to explore how the technique can best be adapted to other populations and domains[4].

## Methods

Sixty-four key informant interviews were conducted with a range of cancer care providers (e.g. medical/radiation/surgical oncologists, nurses and radiation therapists) and senior cancer system administrators within four regions of Ontario, Canada. All interviews were conducted in-person and scheduled for one hour. Ethics approval was obtained from the University of Toronto's Research Ethics Board, with all key informants providing written informed consent prior to participating in the interview.

An interview guide was developed that drew on Tuohy's conceptual work[18] to examine three key inter-related factors of interest in the study: the extent and strength of clinical accountability relationships, the availability and perceived quality of performance information and the structure and allocation of financial incentives. Each interview began with the use of two diagramming methods to explore the complex and multifarious clinical accountability relationships in the Ontario cancer system. Following the use of the diagramming methods, the interview shifted to a traditional semi-structured verbal format to address questions related to performance information and financial incentives. Interviews were recorded, transcribed and analyzed using NVivo 7. Each completed diagram was digitally scanned and key components/relationships were tabulated to facilitate development of summary diagrams for each region and clinical area.

Three diagrams were ultimately presented to interviewees in the same order: Diagram A (participatory diagram – blank sheet); Diagram B (graphic elicitation – macro perspective); and Diagram C (graphic elicitation – micro perspective). The assumption was made that presenting interviewees with a 'blank canvas' (Diagram A) first would have minimal effect on the response to the researcher-prepared diagrams (Diagrams B/C), whereas this assumption would not have applied if the opposite order was used, as providing interviewees with graphic elicitation diagrams before participatory diagramming would have unavoidably influenced their responses. Randomizing the two diagramming methods to different interviews is perhaps the methodologically preferable option for comparative purposes, however this assumes that the two methods are interchangeable rather than potentially complementary and does not address concerns that using only one method per interview could limit our ability to collect useful data to support the study's broader objectives.

**Figure 1:**
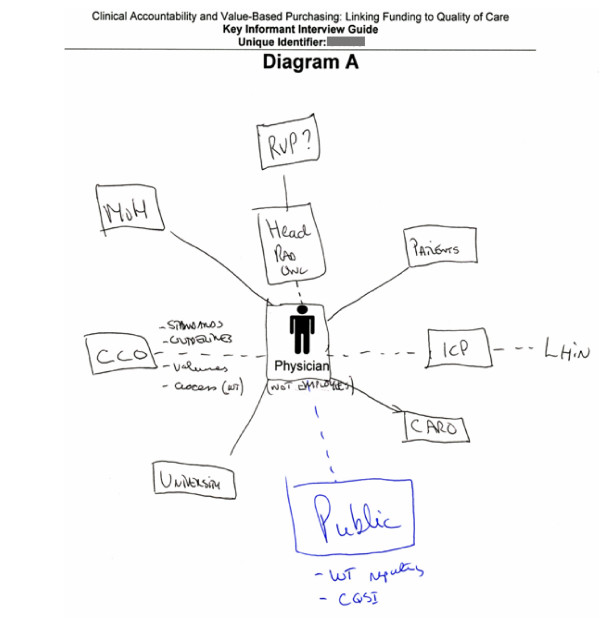
**Example of participatory diagramming**. Diagram A (participatory diagramming) created by an interviewee with black ink pen. Blue ink indicates additional edits made to Diagram A after viewing Diagrams B/C (graphic elicitation).

Diagram A (participatory diagramming) was based on a blank sheet with only a figure representing a physician in the middle. Interviewees were asked to sketch out the types of clinical accountability relationships that oncologists (either medical, radiation or surgical) have in the cancer system and factors that influenced oncologists' clinical decision-making (Figure 1). Since the interview was being recorded, the interviewer reiterated any visual cues used in their explanation, such as pointing at elements that would otherwise not be recorded. To minimize biases, the interviewer did not use verbal prompts to assist the key informant in their diagramming; instead participatory diagramming was used to get an initial snapshot of the informant's perspective at the start of the interview.

**Figure 2:**
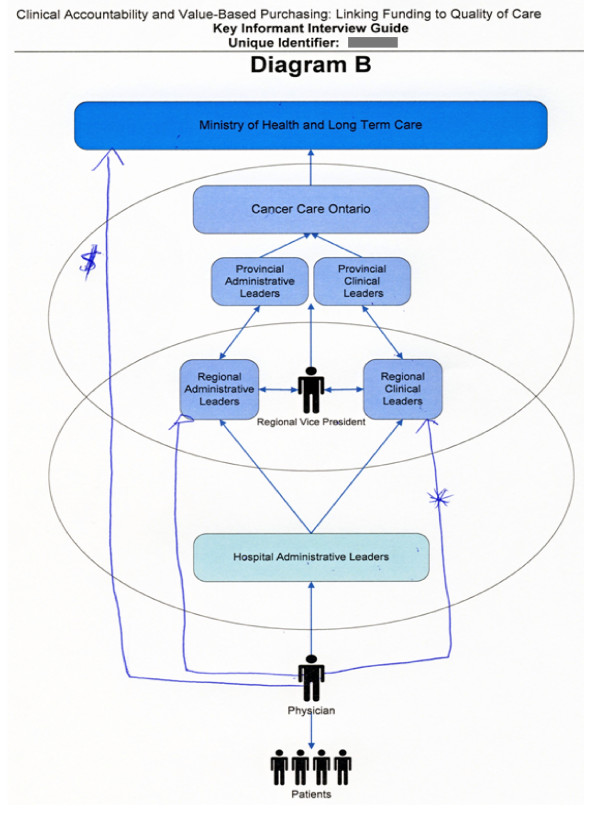
**Example of graphic elicitation (macro perspective)**. Diagram B (graphic elicitation) depicted a macro view of clinical accountability relationships. The interviewee's edits were captured in blue ink.

**Figure 3:**
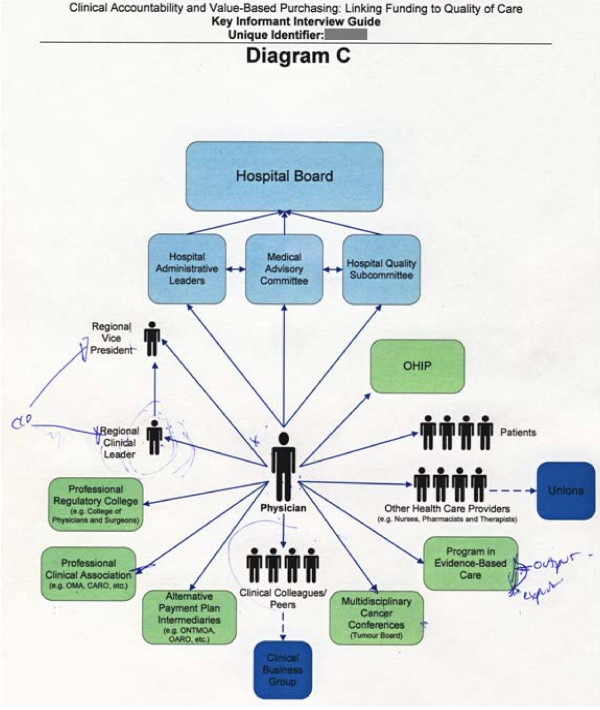
**Example of graphic elicitation (micro perspective)**. Diagram C (graphic elicitation) depicted a micro view of clinical accountability relationships. The interviewee's edits were captured in blue ink.

When interviewees could not add anything further to Diagram A, the interviewee was then presented with the graphic elicitation diagrams. The two researcher-prepared diagrams (derived from a literature review and from document analyses) were designed to represent a macro (Diagram B) and micro (Diagram C) view of clinical accountability relationships. While these two diagrams could have been combined into one larger diagram, the segmentation of complex structures or frameworks into multiple diagrams which are sequentially revealed is one way to help interviewees to focus their attention[3]. As the interviewees included both clinicians and administrators with varying responsibilities and perspectives, we anticipated individual interviewees would be able to relate and react to at least one of the macro or micro diagrams. Therefore, we viewed Diagrams B and C (Figures 2 and 3) as one segmented diagram that facilitated the collection of data from interviewees. Following the suggestions of Crilly et al[4], the interviewer emphasized that the diagrams were 'works in progress' and included the purposeful use of basic shapes, arrows and colours to represent clinical accountability relationships within the Ontario cancer system. Interviewees were asked to comment on the potential accuracies and inaccuracies of each diagram as they related to clinical accountability and decision making, and were encouraged to edit and/or add to the diagrams.

After adapting Diagrams B/C (graphic elicitation), interviewees were encouraged to return to Diagram A (participatory diagramming), if they desired, to make additional edits. Interviewees were provided with a black ink pen when initially responding to Diagram A, and then were provided with a blue ink pen when responding to Diagrams B/C. This facilitated the tracking of the edits and adjustments made after the interviewee's contemplation and use of the researcher-prepared graphic elicitation diagrams.

Our literature review identified potential strengths and weaknesses of each diagramming method[4,5,7,17]. Based on this, measures reflecting two key criteria were developed to assess the utility of diagramming methods in interviews. The first criterion related to the efficiency of the diagramming method for collecting data (i.e., time/effort required to collect data from interviewees). Quantitative efficiency measures included the proportion of interview time required to complete each diagram, the number of interviewee questions and comments regarding the completion of each diagramming method, and the ability of the interviewee to complete the task as instructed. Qualitative observations, including the nature of verbal and written comments regarding relationships and accountabilities elicited as well as the types of questions and/or perceived problems completing the diagrams were also documented and contributed to the assessment of diagramming efficiency.

The second criterion related to the effectiveness of the diagramming method in producing useful data (i.e., collecting relevant, detailed responses from interviewees). Quantitative effectiveness measures included the number of positions/organizations and associated relationships physically or verbally added to the diagram and/or modified by interviewees. This included documenting unique information collected by counting the number of positions/organizations and relationships identified by the interviewee that were not included in our researcher-prepared diagrams (Diagrams B/C). Qualitative observations complemented the quantitative measures, focusing on the level of detail and insight elicited from interviewees through each diagramming method. Each diagram and transcript were examined together to assess the usefulness of interviewees' verbal responses (e.g., where specific examples were provided that helped us to understand which clinical accountability relationships were important and how those relationships were operationalized).

## Results

Data on the efficiency and effectiveness of the participatory diagramming and graphic elicitation methods employed are described below and summarized in Table 1 (efficiency) and Table 2 (effectiveness).

**Table 1:** Assessment of diagramming efficiency

	**Measure**	**Participatory ** **Diagramming** **(Diagram A)**	**Graphic ** **Elicitation** **(Diagrams B/C)**
**1**	**Percentage of interviewees with explicit questions regarding instructions for completing diagramming method**	31% (20/64) Sample interviewee quotations: "You want me to write it down?" "I can just write words?" "Do I have to draw people?" "You want like a map?" "This is supposed to be a flow-chart?" "Does the drawing have to end with me?" "Do you want me to talk as I am doing it?"	17% (11/64) Sample interviewee quotations: "Am I allowed to write on this?" "Do you want me to write on here?" "I can scribble all over it?" "Do you want me to look from up to down?" "So you want me to just start anywhere?"

**2**	**Percentage of interviewees making explicit comments regarding discomfort with diagramming method**	39% (25/64) Sample interviewee quotations: "I don't know how I would begin to draw the picture...I'm not very visual." "I probably haven't done the systematic particularly well." "It's hard to think right on the spot like this."	9% (6/64) Sample interviewee quotations: "I don't know how to explain this to you to get it right." "Does that help? This is tricky." "I didn't make that diagram very well for you." "Does that make sense?"

**3**	**Percentage of interviewees making explicit comments regarding a positive experience with diagramming method**	0% (0/64)	5% (3/64) Sample interviewee quotations: "Interesting exercise, very interesting." "I enjoyed this. This is fun" "Hmmm, this is interesting!"

**4**	**Percentage of interviewees able to complete task physically as instructed**	84% (54/64) The 16% (10/64) interviewees who did not create a diagram each made a point form list instead.	91% (58/64) made physical changes to Diagram B or C. 56% (36/64) made physical changes to both Diagram B and C. The 9% (6/64) interviewees who did not make physical changes to the diagrams each made verbal comments reflecting edits that should be made to the diagrams.

**5**	**Percentage of total interview time spent completing diagramming tasks (mean (range))**	12% (3–28%)	32% (2–59%)

**Table 2:** Assessment of diagramming effectiveness

	**Measure**	**Graphic ** **Elicitation** **(Diagrams B/C)**	**Participatory ** **Diagramming ** **(Diagram A)**
**6**	**Number (mean (range)) of positions/organizations added to diagram physically and verbally**	*Physical: *7 (1–15) *Verbal: *0 (0–7)	*Physical: *2 (0–11) *Verbal: *1 (0–8)

**7**	**Number (mean (range)) of unique positions/organizations added to diagram physically and verbally – excluding the 23 unique positions/organizations presented on Diagrams B/C**	*Physical Unique: *4 (0–9) *Verbal Unique: *0 (0–3)	*Physical Unique: *1 (0–9) *Verbal Unique: *1 (0–5)

**8**	**Number (mean (range)) of relationships (arrows/lines) added to diagram physically and verbally**	*Physical: *7 (0–20) *Verbal: *1 (0–11)	*Physical: *3 (0–18) *Verbal: *2 (0–12)

**9**	**Number (mean (range)) of unique relationships (arrows/lines) added physically and verbally – excluding the 33 unique relationships presented on Diagrams B/C**	*Physical Unique: *4 (0–12) *Verbal Unique: *1 (0–6)	*Physical Unique: *3 (0–16) *Verbal Unique: *2 (0–12)

**10**	**Number (mean (range)) of relationships (arrows/lines) modified physically and verbally**	*n/a*	*Physical: *3 (0–12) *Verbal: *3 (0–12)

**11**	**Percentage of interviewees returning to Diagram A after viewing Diagrams B/C**	17% (11/64) returned	*n/a*

**12**	**Number (mean (range)) of additions/edits made to Diagram A after viewing Diagrams B/C**	Positions/Organizations added/edited upon return to Diagram A: 1 (0–2) Relationships added/edited upon return to Diagram A: 1 (0–4)	*n/a*

	**Qualitative observations**	Verbal comments accompanying diagrams captured greater breadth of response reflecting information not identified in pre-interview review work.	Verbal comments accompanying diagrams focused on researcher-prepared items, with responses including more details, insights and examples.

### Diagramming efficiency

Participatory diagramming generated more explicit questions and comments regarding the instructions for completing the diagramming method than graphic elicitation (Measure 1) as well as more explicit comments regarding participant discomfort (Measure 2). While participatory diagramming brought forth a greater number of questions and comments, the content was similar for both methods. Regarding the instructions for both methods, participants sought reassurance about physically drawing on the diagrams. In response to Diagram A (participatory diagramming), participants wanted to confirm that they could draw the diagram as they saw appropriate (both in regards to content and format) and when completing Diagrams B/C (graphic elicitation), participants' questions and comments focused on confirming that they should make physical changes to the diagrams, not just verbal ones. While there were fewer comments regarding participant discomfort for graphic elicitation, they were of similar content. Participants expressed concern about 'thinking on the spot', often reporting the feeling that they were being tested and apologizing for poor drawing and diagramming skills. The only explicitly positive comments made about the diagramming methods were specifically with respect to graphic elicitation (Measure 3). No comparable positive comments were made about participatory diagramming.

Despite some expressed concerns about the diagramming methods, most participants were able to complete the diagramming task as instructed for both methods (Measure 4). The majority of participants were able to create some form of a diagram using boxes, labels, arrows and/or lines. The 10 (16%) participants who chose not to create a diagram made point-form lists on the paper instead. For graphic elicitation, participants were all instructed and encouraged to make physical edits to the prepared diagrams (Diagrams B/C), and generally were able to do so for at least one of the graphic elicitation diagrams. Those who did not make physical edits on the diagrams verbally commented on them and these comments were captured by audio recording.

The time required to explain the method and for the interviewee to complete the exercise is an important efficiency measure, particularly for interviews involving elite informants who face intense demands on their time, as was the case in this study. The interviews were scheduled for one-hour time slots and averaged 65 minutes with a range of 36 to 107 minutes. Participatory diagramming took up a smaller percentage of the total interview time compared with graphic elicitation (Measure 5). Only two (3%) interviewees spent more time on Diagram A (participatory diagramming) than they did on Diagrams B/C (graphic elicitation).

### Diagramming effectiveness

On average, interviewees physically added more positions/organizations to Diagram A (participatory diagramming) than to Diagram B/C (graphic elicitation) (Measure 6). This was expected given that Diagram A (participatory diagramming) involved a blank sheet with only a figure of a physician included. It therefore required interviewees to create their diagram from scratch, while Diagrams B/C (graphic elicitation) included 23 existing positions and organizations. However, when considering the addition of unique positions and organizations beyond the 23 that were included as a stimulus in Diagrams B/C, Diagram A (participatory diagramming) still produced more unique information than Diagrams B/C (graphic elicitation) (Measure 7). A similar outcome was seen when looking at the number of relationships (e.g., arrows and lines connecting different positions and organizations) physically added to the diagrams. As expected, Diagram A (participatory diagramming) again had a higher mean number of relationships added (Measure 8). However, the mean number of unique relationships (e.g., not identified in Diagrams B/C) added were similar for each diagramming method (Measure 9).

We also documented interviewees' specific verbal additions of positions/organizations and relationships in response to both diagramming methods that were not captured physically on one of the diagrams. While, Diagrams B/C (graphic elicitation) generated more verbal additions than Diagram A (participatory diagramming), the difference between the two methods was minimal (Measures 6–9).

In addition to adding new positions/organizations and new relationships to Diagrams B/C (graphic elicitation), participants were also encouraged to make edits to the researcher-prepared relationships presented. These edits included specific indications regarding where boxes should be moved, using solid vs. dotted lines to indicate the strength of a relationship, reversing arrow heads to indicate the principal driver of a relationship and ranking or weighting the relative importance of each position/organization to oncologists. While not directly comparable to participatory diagramming, these physical edits to the graphic elicitation diagrams were important as they captured additional information on the nature of clinical accountability relationships (Measure 10).

By switching the pen ink colours when introducing the graphic elicitation section, we were able to track additions or edits participants made to Diagram A (participatory diagramming) after being exposed to Diagrams B/C (graphic elicitation). Only 11 (17%) interviewees returned to their initial Diagram A after responding to Diagrams B/C (Measure 11). Those that did return to Diagram A made only minimal additions/edits to the positions/organizations and relationships they had already represented in their initial diagram (Measure 12).

While the physical and specific verbal additions/edits to the diagrams provided important information on the existence and structure of clinical accountability relationships, it was the additional verbal comments accompanying these additions/edits to Diagrams B/C that provided the most detailed information. These comments opened up the black box to provide insights on the relative importance of various clinical accountability relationships and how these relationships were operationalized in the context of the Ontario cancer system. Most interviewees used the researcher-prepared diagram elements as talking points from which they provided assessments specific to each element, including examples that elucidated how these relationships worked. For example, two quotations from interviewees reflected the nature of the insights obtained using graphic elicitation. In response to Diagram B, a clinical leader described the accountability relationship with the provincial cancer agency:

"The relationships with CCO [Cancer Care Ontario] are stronger now, I think, in terms of accountability, certainly than they were in the past, even when we [oncologists] were employees of CCO. The accountability for patients has always been there...but the accountability for seeing 'X' number of patients has changed and that's come with the APP [alternate payment plan], with the accountability for wait times and getting them shorter...so that if you were to take away CCO, you'd take away a lot of the accountability..."

Another oncologist, in response to Diagram C, provided insight on the accountability relationships between oncologists and their clinical colleagues/peers and department heads:

"...as a medical oncologist I will report directly to my colleague within medical oncology or within medical staff meetings...without necessarily going through the head of the department, and that maybe reflects the fact that I'm a staff physician, I'm working with them all the time... But generally speaking if there's something that I want done I'll try to get the head involved to do it..."

The data collected through the graphic elicitation diagrams allowed us to understand more fully the relative importance and influence of particular accountability relationships on oncologists.

## Discussion

Despite a few questions or concerns expressed by participants, we found that interviewees, overall, had few problems responding to either participatory diagramming or graphic elicitation methods. Given that our sample included a wide range of positions from clinicians to senior health care administrators, our results suggest that both methods are appropriate with populations that have no particular experience with the production or interpretation of diagrams. Even when participants were not able to create or edit a diagram as explicitly instructed, useful information was still collected through point-form lists and verbal comments.

For our project, participatory diagramming (Diagram A) offered some unique advantages over graphic elicitation (Diagrams B/C). As we did not use prompts in our application of participatory diagramming, the data collected represented the relatively unbiased views and perspectives of the interviewees on the topic. Participatory diagramming required interviewees to think broadly about who oncologists interact with, capturing more unique relationships and positions/organizations (not identified through our literature review or document analysis and therefore not depicted in Diagrams B/C). Participatory diagramming produced more graphic rather than verbal responses resulting in a more useful graphic data source (the resulting diagram) while taking less time for interviewees to complete than graphic elicitation. However, in comparison to graphic elicitation, interviewees' verbal responses accompanying participatory diagramming tended to provide less detailed commentary.

Graphic elicitation focused the interviewees' attention to issues that we were particularly interested in. As the researcher-prepared diagrams contained a fairly large number of positions/organizations and relationships among them, graphic elicitation resulted in less physical additions or edits made to the diagrams. However, the verbal comments accompanying the graphic elicitation section of the interview were more detailed and insightful than verbal comments accompanying the participatory diagramming section. Graphic elicitation generated verbal comments that included reflections and critiques on physician accountability relationships and offered detailed examples of the nature of these interactions that were helpful to answering important questions of the broader study. While the researcher-prepared diagrams were effective tools for eliciting high-quality verbal comments from interviewees, this section of the interview also required considerably more time to complete than anticipated, reducing the interviewer-interviewee interchange for subsequent sections of the interview.

Overall we found participatory diagramming to be the most efficient method to extract relatively unbiased data in a graphical form and produce important unique data not obtained through graphic elicitation. Graphic elicitation consumed a lot of valuable interview time, impacting on our ability to collect data on other important interview questions, however it was more effective in producing in-depth consideration of key, researcher-identified issues. This balance between the efficiency and effectiveness of the two diagramming methods is at the crux of the methodological decision regarding the optimal use of diagramming in interviews.

So how would we integrate diagramming methods into our research design if we were to undertake the process again? With the benefit of this analysis, we see value in incorporating both participatory diagramming and graphic elicitation methods with conventional interview techniques. Our intent would be to maximize the efficiency of participatory diagramming as a tool to collect a quick snapshot of interviewees' views by avoiding the use of verbal prompts and providing only limited direction to interviewees through the exercise. We would again follow with graphic elicitation, but with a simpler diagram with a limited number of researcher-prepared items that we would want interviewees to respond to and then more assiduously direct interviewees through the diagram to reduce the time required to complete the diagram. Together, participatory diagramming and graphic elicitation methods represent complementary tools that can enhance data collected through qualitative interviews.

The findings of this study need to be considered in light of some key methodological limitations. Given the broader requirements of our study, both diagramming methods were employed, in the same order (participatory diagramming followed by graphic elicitation), in all key informant interviews. Therefore, no control group was used to compare the efficiency/effectiveness of conventional qualitative interviewing methods when diagramming methods were not employed. We encourage further comparative research to examine the various strengths and weaknesses of using diagramming methods in qualitative interviews identified in this study. This should ideally include four distinct comparison groups of qualitative interviews, employing: (1) conventional interview methods but no diagramming methods, (2) both participatory diagramming and graphic elicitation methods, (3) participatory diagramming method only, and (4) graphic elicitation method only.

## Conclusion

We are not aware of other studies that have directly assessed both diagramming methods or applied either method to a large sample of clinicians and health care administrators. Subject to the methodological limitations of our study, we believe these findings offer guidance for those considering the use of diagramming methods for qualitative interviewing. Our application and subsequent assessment of the two methods revealed particular strengths and weaknesses. We found participatory diagramming to be an efficient method for collecting data in a graphic form, but may not generate the depth of verbal response that many qualitative researchers seek. Graphic elicitation was more intuitive, better understood and preferred by most respondents, and often provided more contemplative verbal responses, however this greater depth was achieved at the expense of more interview time. Qualitative researchers considering the use of these diagramming methods in interviews need to consider this effectiveness-efficiency balance. Efforts to maximize the inherent strengths and minimize the inherent weaknesses of each method should enhance their application, whether they are used in combination or separately.

## Competing interests

The authors declare that they have no competing interests.

## Authors' contributions

All authors participated in the conception and design of the study. The key informant interviews were conducted by MJD and LLC. MJU conducted the diagram analysis and prepared the original draft of the manuscript. All authors reviewed and critically revised the original and subsequent manuscript drafts, and approved the final manuscript.

## Pre-publication history

The pre-publication history for this paper can be accessed here:


